# The Effects of Environmental Factors on the Efficiency of Clinical Commissioning Groups in England: A Data Envelopment Analysis

**DOI:** 10.1007/s10916-017-0740-5

**Published:** 2017-05-09

**Authors:** Rumbidzai Takundwa, Sue Jowett, Hugh McLeod, Maria Cristina Peñaloza-Ramos

**Affiliations:** Health Economics Unit, Institute of Applied Health and Research, University of Birmingham, Birmingham, B15 2TT UK

**Keywords:** Data envelopment analysis, Efficiency, Primary care, Clinical commissioning group

## Abstract

Clinical Commissioning Groups (CCGs) were created in 2013 to make the NHS more responsive, efficient and accountable. A large number of different indicators can be used to measure the quality and outcomes of services provided by CCGs, however there is currently no single measure of overall efficiency available. The performance of CCGs may also be confounded by environmental factors such as deprivation, population size and burden of disease. Data Envelopment Analysis (DEA) is a linear programming technique that can be used to measure the relative efficiency of a given set of organisations. To use DEA to measure the efficiency of English CCGs and assess the impact of environmental factors. This study estimates the technical efficiency of 208 CCGs in England using DEA. The inputs and outputs used include budget allocation, number of general practitioners, mortality rates, patient satisfaction and Quality and Outcomes Framework achievement scores. Regression analysis is used to assess the effects of environmental factors on efficiency, such as population size, prevalence of disease, and socio-economic status. Twenty-three percent (47/208) of CCGs were efficient compared to the others. Three environmental factors were statistically significant predictors of efficiency: CCGs with smaller population sizes were more efficient than those with larger ones, while high unemployment rates and a high prevalence of chronic obstructive pulmonary disease led to a decrease in efficiency scores. Comparative deprivation was not a significant predictor of efficiency. The finding that the relationship between deprivation and efficiency is not statistically significant suggests that NHS England’s adjustment for environmental factors within the CCG-level budget allocation is broadly successful. This study shows the potential of DEA for assessing technical efficiency at CCG-level in the English NHS.

## Introduction

The National Health Service (NHS) in the UK delivers universal tax-funded healthcare to the population, free at the point of delivery. Within the current structure of the NHS in England, about two thirds of the £106 billion NHS budget is allocated to Clinical Commissioning Groups (CCGs), which are responsible for the commissioning of healthcare services including secondary care, rehabilitation services and mental health services to their local population [1]. In allocating funds to CCGs, NHS England considers several factors including population size, geographical variation in the costs of providing healthcare, population age structure and other socio-economic factors [2]. However, there is considerable discretion about how funds are used, which has led to concern about unwarranted variation in activity and the need to improve efficiency [1]. The current focus on efficiency is also driven by the sustained period of financial austerity in which the NHS is operating [3].

CCGs are membership organisations, representing general medical practices within geographical areas, which design, plan and commission local health services to meet the needs of their populations [4]. In 2013, 211 CCGs were created through the Health and Social Care Act (2012) replacing 152 Primary Care Trusts (PCTs). The reforms brought about by the Act were intended to make the NHS more responsive, efficient and accountable [4]. Measures relating to the quality of health services provided by CCGs and associated health outcomes are published in the CCG Outcomes Indicator Set collected by NHS Digital, formerly known as the Health and Social Care Information Centre (HSCIC) [5]. The indicator set includes a range of data on different measures of quality and outcomes including emergency hospital admissions, mortality rates, and cancer survival rates. However, it does not provide a single measure of overall or comparative efficiency across all indicators at CCG-level.

Data envelopment analysis (DEA) is one method that can be employed to measure efficiency, particularly when comparing the efficiency of organisations that use multiple inputs to produce multiple outputs as is the case in healthcare [6]. Using DEA, this paper aims to estimate the comparative efficiency of English CCGs and assess the impact of environmental factors on efficiency scores using regression analysis.

## Methods

DEA is a linear programming technique that is concerned with measuring the relative efficiency of a given set of organisations, entities or programmes which are referred to as “Decision Making Units” (DMUs) [6]. It assumes that the DMUs produce homogenous outputs using the same inputs and provides a way to compare them against each other. It has been applied in many different industries and activities in different countries and contexts [7]. It is computed by the following:

$$ { \max\ \mathrm{h}}_0=\frac{\sum_{r=1}^s{u}_r\ {y}_{r0}}{\sum_{i=1}^m{v}_i\ {x}_{i0}} $$

Subject to

$$ \frac{\sum_{r=1}^s{u}_r\ {y}_{r j}}{\sum_{i=1}^m{v}_i\ {x}_{i j}}<1; j=1,\dots \dots \dots .. n $$

$$ {\mathrm{V}}_{\mathrm{r},}{\mathrm{u}}_{\mathrm{i}\ }\ge 0;\mathrm{r}=1\dots \dots \dots \dots \dots \mathrm{s}:\mathrm{i}=1\dots \dots \dots \dots .\mathrm{m} $$

where *y*_*rj*_ and *x*_*ij*_ are the known outputs and inputs of the jth DMU respectively and *u*_*r*_ and *v*_*i*_ are the weights assigned to the inputs and outputs [6, 7]. The weights take a positive value with each DMU having its own set of weights. Each DMU is given the most favourable weighting that constraints allow. The model illustrated above is output-orientated because it shows the maximum output that could be achieved for a given level of input if a DMU is operating efficiently. The inverse of the model would be input-orientated and it would allow for the estimation of the minimum level of input required to produce a level of output if operating at full efficiency. The original model assumes constant returns to scale (CRS). However, it was later extended to include a model that could accommodate variable returns to scale [8].

DMUs with efficiency scores of 1 (i.e. those exhibiting best practice) form a frontier or envelopment surface and any DMUs operating inefficiently will lie below the frontier. This is similar to the production possibility frontier used in economic theory. However, unlike the production functions used in economics, DEA frontiers are derived from empirical data and not economic theory. The constant returns to scale model is only appropriate for use where it is known that the DMUs are operating at an optimal scale. In many cases, there may be diminishing or increasing returns to scale, and it is necessary to use the variable returns to scale model.

DEA has some advantages and disadvantages over other methods of measuring efficiency such as stochastic frontier analysis (SFA) and corrected ordinary least squares (COLS) cost frontier analysis [9]. Mainly, it does not require any prior assumptions about the specification of cost/production functions and standard errors unlike econometric methods.

This study aims to use DEA to measure the efficiency of English CCGs and assess the impact of environmental factors. The decision-making units of concern in this study are English CCGs. Data for each CCG were collected for the financial year 2014/15 and in cases where this was not available, data for the 2014 calendar year were used. The main sources of data were NHS Digital (formerly HSCIC) and NHS England (as shown in the footnotes to Table 1). In all, data for 20 variables were collected, 2 of which were input variables, 7 were output variables and 11 were environmental variables.

**Table 1:** CCG-level descriptive statistics for input and output variables

Variable name	Mean	Median	Standard deviation	Min	Max
INPUTS					
Per capita CCG funding allocation (£000 s)^a^	1.15	1.15	0.08	1.00	1.50
Number of GPs per 100,000 population^b^	61.6	61.1	8.6	41.6	90.0
OUTPUTS					
Directly standardised average health status score for individuals aged 18 and over ^c^	0.7	0.7	0.0	0.6	0.8
Directly age and sex standardised respiratory disease survival rate per 100,000 population^d^	28.8	26.6	9.6	11.0	61.0
Directly age and sex standardised cancer survival rate per 100,000 population^d^	122.8	121.5	17.0	84.1	171.9
QOF cardiovascular disease score^e^	8.9	9.0	0.7	5.8	10.0
QOF cancer score^f^	10.8	10.9	0.2	8.9	11.0
QOF COPD score^g^	33.7	33.9	1.0	28.3	35.0
Percentage of patients who would recommend the GP practice to others^c^	77.2	77.5	5.0	59.7	91.3

^a^Source: [12]

^b^Source: [13]

^c^Source: [14] Health-related quality of life measured using EQ-5D-5 L, where 0 is dead and 1 is full health

^d^Source: [5] 1 – directly age and sex standardised mortality rate from respiratory disease/ cancer (mean normalised)

^e^Source: [15] The average achievement score per practice out of a maximum of 10, for patients between the ages of 30 and 74 newly diagnosed with hypertension, who have a recorded risk assessment score and who are currently being treated with statins

^f^Source: [15] The average achievement score per practice out of a maximum of 11, for two measures: patients recorded with a cancer diagnosis since April 2003, and the percentage of patients with cancer, diagnosed within the preceding 15 months, who have a patient review recorded as occurring within 6 months of diagnosis

^g^Source: [15] The average achievement score per practice out of a maximum of 35, for five measures relating to COPD patients, including registration, diagnosis, review, and treatment

The inputs included in this study represent labour and financial inputs, while the outputs include a small selection of the CCG Outcome Indicator Set currently used by NHS Digital to measure the quality of primary care. The outcome indicator selection process began by identifying issues which were leading priorities within the NHS in England [10]. The selected outcome measures were premature mortality, long term conditions and patient satisfaction. The reduction of premature mortality is high on the agenda in the UK [11] and in this paper is assumed to be a key output of the healthcare system. Cancer, respiratory disease and cardiovascular disease are among the top 5 causes of premature mortality in England [11]. A reduction in respiratory disease and cancer mortality was assumed to be a key output of CCG commissioning. Coronary heart disease (CHD) survival was excluded because model stepwise and correlation analysis model selection techniques showed that it added no explanatory power. Additionally, quality and outcomes framework (QOF) achievements in the management of cancer, chronic obstructive pulmonary disease (COPD) and cardiovascular disease were included as short term outputs in these specific disease areas. As mortality is a long term output, it may fail to capture any improvements in the short term, hence the inclusion of QOF outcomes. The proportion of patients who would recommend their general practitioner (GP) practice to others was included as a proxy measure of patient satisfaction. Quality of life of individuals with long term conditions was chosen as an output for the management of long term conditions in the community.

CCGs with missing data on any of the variables were excluded from analysis; this applied to 3 out of a total 211 CCGs in England, thus bringing the total units of analysis to 208. Table 1 shows the descriptive statistics for all variables.

Environmental variables are variables external to the production process that may still affect the utilisation or availability of resources [16]. In this study, economic, social, physical and lifestyle characteristics that are deemed to be wider determinants of health [17–19] and are likely to confound any measure of efficiency have been included. Additionally, some disease prevalence variables have been included, as they may influence disease-specific mortality outcomes. The environmental variables included are a subset of the public health outcomes framework, and were selected on the basis that they reflect deprivation, population demographics, and burden of disease and lifestyle factors on CCG resources. The variables included are by no means an exhaustive list of potential candidates. Table 2 presents the descriptive statistics for the variables used.

**Table 2:** CCG-level descriptive statistics for environmental variables

Variable name	Mean	Median	Standard deviation	Min	Max
Indices of multiple deprivation ranking^a^	N/A	N/A	N/A	1	209
GP Registered population^b^	270,631	236,440	143,917	73,093	905,649
GP registered population aged under 18 years (%)^b^	20.72	20.69	1.97	15.31	30.60
GP registered population aged 65 years and over (%)^b^	17.08	17.50	4.37	5.72	28.31
GP registered population aged 18 and over with a long standing health condition (%)^c^	54.16	54.55	3.94	44.18	63.25
GP registered population aged 18 and over who are unemployed (%)^c^	4.89	4.17	2.36	1.35	15.71
Estimated smoking prevalence (%) ^d^	18.57	18.28	2.85	12.28	27.05
Prevalence of obesity (%)^d^	9.15	9.14	2.09	4.01	14.11
COPD prevalence (%)^d^	1.86	1.82	0.59	0.77	3.72
Cancer prevalence (%)^d^	2.28	2.34	0.53	0.76	3.49
CHD prevalence (%)^d^	3.30	3.40	0.85	1.33	5.21

^a^Source: [20]

^b^Source: [21]

^c^Source: [14]

^d^Source: [15]

All mortality variables were transformed into survival rates by converting the values to their inverse form. Additionally, to address the magnitude imbalance within the dataset, all input and output variables were mean normalised.

DEA models were run using MaxDEA 6.6 software [22]. QGIS software [23] was used to map the results of the final DEA model.

Regression analysis was used to quantify the relationship between efficiency and environmental variables. The efficiency scores computed from DEA were used as the dependant variable and the environmental factors used as explanatory variables. As efficiency scores are limited to values between 0 and 1, Tobit regression was used to estimate the relationship between the scores and environmental variables using Stata 13.1 software [24].

## Results

The average technical efficiency score for all CCGs in the model was 0.92. A total of 47 CCGs had a score of 1 (Table 3). The remaining 161 CCGs were inefficient and had an average score of 0.9. This means that on average, the inefficient CCGs could reduce their inputs by 10% and still produce the same level of outputs if they were operating efficiently. The CCG that was the least efficient compared to others had a score of 0.75.

**Table 3:** Distribution of efficiency scores

Technical efficiency scores	CCGs	
	Number	%
1	47	22
0.940–0.999	49	24
0.890–0.939	46	22
0.844–0.889	39	19
0.750–0.843	27	13

### Geographical distribution

**Map 1:**
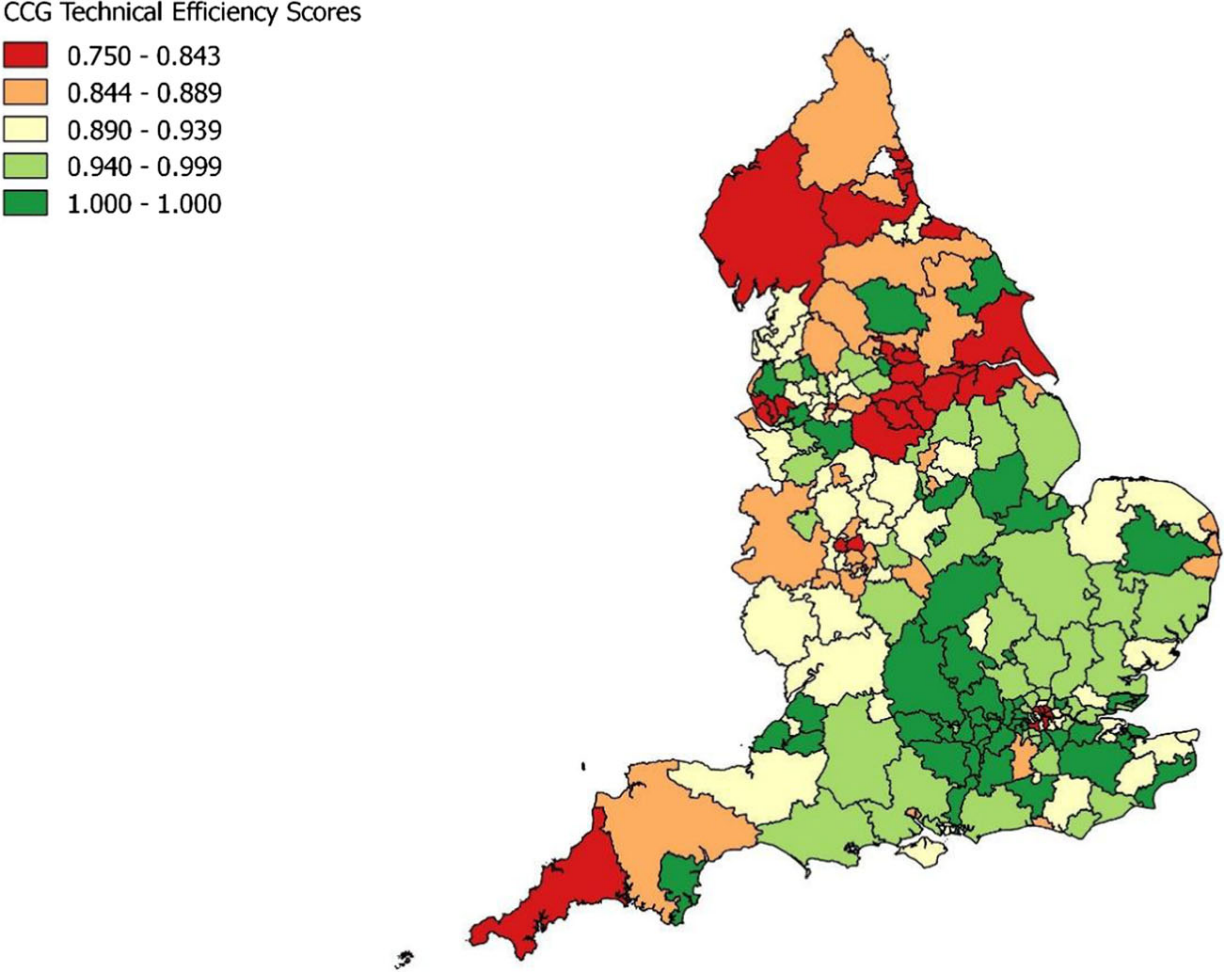
Geographical distributions of technical efficiency scores

The geographical inequalities that exist in health outcomes are well documented [25]. To explore the implications of this geographical variation on the efficiency of CCGs, technical efficiency scores were plotted on a map of England. Map 1 shows that a high number of CCGs with the lowest technical efficiency scores are clustered in Northern England while CCGs with higher technical efficiency scores are mostly in Central and Southern regions of England.

The percentage of CCGs experiencing technical efficiency scores of 1 in each deprivation quintile varied (Table 4). It appears that the most deprived CCG quintile in England had the lowest mean efficiency score and the fewest efficient CCGs compared to the other quintiles (Table 4). This relation is explored further using the Tobit regression model.

**Table 4:** Mean technical efficiency scores by deprivation quintile

Deprivation quintile	Mean technical efficiency score	Efficient CCGs with a technical efficiency score of 1	
		Number	%
1 (most)	0.87	3/42	7
2	0.92	9/42	21
3	0.92	5/42	12
4	0.94	7/42	17
5 (least)	0.98	23/40	57

### Environmental factors that affect the efficiency of a CCG

The results of the Tobit regression model are shown in Table 5. The categorical variable for the population size quintile had negative coefficients on quintiles 2–5 with quintile 1 as the reference. Quintile 1 had the lowest population size, therefore being in a quintile with a higher population caused lower efficiency scores. This relationship was statistically significant for quintiles 4 and 5 at the 5% level but not for quintiles 2 and 3. A 1 % increase in population in quintile 4 is associated with a 5.2% fall in efficiency (Table 5).

**Table 5:** Regression analysis results for predictors of technical efficiency at CCG level

Variable	Coefficient	*p*-value	95% Confidence intervals	
			Lower limit	Upper limit
Deprivation IMD quintile 1 (lowest as reference)				
2	0.008	0.575	−0.022	0.039
3	−0.014	0.459	−0.054	0.024
4	−0.015	0.519	−0.061	0.031
5	0.010	0.723	−0.046	0.067
Population quintile 1 (lowest as reference)				
2	−0.008	0.517	−0.034	0.017
3	−0.019	0.136	−0.044	0.006
4	−0.052	<0.001*	−0.077	−0.026
5	−0.043	0.001*	−0.068	−0.018
GP registered population aged under 18 years (%)	0.016	<0.001*	0.011	0.022
GP registered population aged 65 years and over (%)	0.005	0.179	−0.002	0.013
GP registered population aged 18 and over with a long standing health condition (%)	0.002	0.478	−0.004	0.007
GP registered population aged 18 and over who are unemployed (%)	−0.014	0.001*	−0.022	−0.006
Smoking prevalence (%)	−0.001	0.662	−0.007	0.005
Prevalence of obesity (%)	−0.003	0.447	−0.009	0.004
COPD prevalence (%)	−0.055	0.005*	−0.093	−0.017
Cancer prevalence (%)	−0.030	0.341	−0.093	0.032
CHD prevalence (%)	−0.001	0.967	−0.030	0.029
Constant	0.703	<0.001*	0.456	0.950
Sigma	0.052		0.046	0.058

LR Chi^2^ = 144.14 Prob > chi^2^ = <0.0001

*Statistically significant *p* < 0.05

The coefficient for the proportion of children registered within the CCG had a positive sign and was statistically significant. Higher proportions of under 18 s registered in the practice resulted in higher efficiency scores. The rate of unemployment and prevalence of COPD had the opposite effect, with negative coefficients.

IMD ranking, the proportion of patients aged 65 plus, patients with long term conditions, smoking and the prevalence of obesity, cancer and CHD were not found to be statistically significant predictors of technical efficiency.

## Discussion

Using DEA, this study has computed the technical efficiency scores of 208 English CCGs. The findings indicate that CCGs serving smaller populations appear to be more efficient. This goes against the a priori assumption that CCGs with larger populations would be able take advantage of economies of scale, and therefore be more efficient than smaller ones. When looking at the geographical distribution of efficiency scores, there appeared to be a negative relationship between deprivation and efficiency scores. However, regression analysis showed that the relationship at CCG-level was not statistically significant.

A further a priori assumption was that the burden of disease (i.e. the prevalence of long term conditions, CHD, COPD and cancer) would have a negative impact on the efficiency of CCGs due to the increased demand for services. Regression analysis showed that this anticipated impact, whilst there, was not statistically significant. The only exception was the prevalence of COPD, which had a significant negative relationship with technical efficiency scores. Lifestyle factors such as smoking and obesity were also expected to increase the burden of disease and lead to lower efficiency scores, however regression analysis showed that there was no statistically significant link. Having high proportions of unemployed residents led to a statistically significant increase in inefficiency. This was not surprising as individuals from low socioeconomic groups are known to experience less favourable health outcomes than more affluent groups. The prevalence of COPD and the rate of unemployment were found to be statistically significant predictors of efficiency. These can be improved through targeted public health interventions to reduce smoking and other lifestyle factors as well as economic investment in affected areas.

This study is the first to use DEA to quantify the efficiency of CCGs in England, and assess environmental factors that impact on efficiency. However, the study has a number of limitations. Firstly, the DEA programme has been allowed full flexibility in assigning weights to input and output variables. As DEA methods aim to paint a DMU in the best light possible, some inputs may be completely ignored (i.e. given a weight of 0) for some DMUs to make them as efficient as possible.

Another limitation was the unavailability of data for particular variables which ideally would have been included in the analyses. For example, the quality of hospital infrastructure’s at CCG level i.e. a new 100 bed hospital is expected to be much better placed than a Victorian 100 bed hospital to provide services. Several environmental variables were obtained from surveys and may not be a true representation of the population. This means that estimated relationships may be constrained by the accuracy of available data. It is possible that some important variables are missing from the models and that some unnecessary variables have been included.

This study found average efficiency scores that are similar to those of previous studies in the UK at the primary care administrative body level. Salinas-Jimenez and Smith [26], Giuffrida and Gravelle [27] and Martin and Smith [28] found that inefficient units had average efficiency scores of 0.93, 0.99 and 0.90 respectively. This study is also comparable to previous studies in its findings around environmental variables. Martin and Smith [28], using a Tobit regression similar to the one in this study, found that deprived PCTs were significantly less efficient than those with lower levels of deprivation. In contrast to this study, they did not find an association between population size and efficiency scores. Varela et al. [29] found the population size of municipalities in Brazil had a strong link to their technical efficiency in delivering healthcare, with smaller municipalities found to be more efficient than larger ones.

This study’s finding that the relationship between deprivation and efficiency is not statistically significant suggests that NHS England’s efforts to adjust for environmental factors, such as those used here, are broadly reflected within the CCG-level per capita budget allocation. The budget allocation formula aims to adjust for comparative need and unavoidable differences in costs for providers associated with their geographical location (known as the market forces factor) [30].

The Health and Social Care Act (2012) took public health departments out of NHS PCTs and placed them within local authorities. It is thought that local authorities are better placed to identify the broader needs of the population that impact on public health. Whether this will lead to improvements in the factors that affect CCG-level efficiency remains to be seen. Additionally, as the economy slowly recovers from the 2008/9 recession, unemployment rates are expected to decrease. However, this study suggests that policies designed to raise employment rates in disadvantaged areas are important.

Further research on appropriate measures of output in healthcare would be valuable to build on these results. Additionally, the development of guidelines on measuring efficiency in healthcare would promote robust methodological processes and enhance comparability across studies.
